# Split-Mouth Comparison of Pain and Healing Associated With the Electrocautery and Bur Abrasion Methods in the Treatment of Depigmentation: A Case Report

**DOI:** 10.7759/cureus.59455

**Published:** 2024-05-01

**Authors:** Yogita Vasyani, Subodh Gaikwad, Chitrika Subhadarsanee, Anup Shelke, Khushboo Durge

**Affiliations:** 1 Department of Periodontology, Dr. Hedgewar Smruti Rugna Seva Mandal's Dental College & Hospital, Hingoli, IND; 2 Department of Periodontology, Sharad Pawar Dental College, Datta Meghe Institute of Higher Education and Research, Wardha, IND

**Keywords:** soft tissue trimming bur, melanin pigmentation, bur abrasion, electrocautery, depigmentation

## Abstract

The most common aesthetic issue that affects people's smiles is gingival pigmentation, especially in those with high smile lines. This pigmented gingiva is thought to be naturally occurring melanin pigments that add to the gingiva's endogenous pigmentation. The goal of plastic periodontal surgery known as "gingival depigmentation" is to lighten the dark gingiva by scraping off the gingival epithelium. Gingival depigmentation has been performed with a variety of techniques, including bur abrasion, scraping, partial thickness flap, cryotherapy, electrocautery, and laser. The present case comprised a split-mouth design in which depigmentation using an electrosurgical unit and soft tissue trimming bur was used for the maxillary sections, and evaluated the difference in pain felt by the patient and healing of the surgical site between the sites treated with the electrosurgical unit and bur abrasion method. A visual analog scale (VAS) was used to quantify pain felt by the patient on the seventh day, whereas healing was assessed on the seventh day and at a one-month interval visually. The results of this study showed that the electrocautery-treated site showed better results in terms of pain experienced by the patient and also with the surgical site healing.

## Introduction

The gingival tissue plays a role in determining the harmonious relationship of the natural smile, in addition to the gingival position, color, and form of the teeth [1]. Patients with a high smile line start to think about their pigmentation from an aesthetic standpoint. Gingival health ratings range from light pink to dark red or violet. Skin becomes dark due to a naturally occurring chemical called melanin pigmentation [2]. Every race has gingival melanin pigmentation [3]. The pigmentation of oral melanin is thought to be multifactorial, resulting from a range of physiological or pathological factors that can be localized or systemic in nature. These include genetics, tobacco consumption, and long-term use of different medications, mostly antimalarial agents, and tricyclic antidepressants [4]. There is no preference for oral pigmentation based on race or gender. Within and between races, as well as between individuals belonging to a similar race but with different regions of a similar mouth, there are differences in the allocation and severity of racial gingival melanin pigmentation of the oral cavity.

The desire of the patient for a better appearance is the main indication for undergoing depigmentation. Technique selection should always rely on individual preferences and clinical background, as these factors yield better outcomes [2]. The patient's request for cosmetic enhancement serves as the first sign that the procedure is necessary [5].

Gingival melanin hyperpigmentation can be treated with an ample number of techniques, which show varying degrees of success, including gingivectomy done using free gingival autografts and acellular dermal matrix allografts, electrocautery, cryosurgery technique, soft tissue trimming bur abrasion therapy, and laser technique [6].

Electrocautery is an effective method of gingival depigmentation. Re-pigmentation is less likely when electrocautery is used because it significantly slows down the melanin cell migration from locally situated cells. There are numerous benefits, including the reduction in the bleeding tendency and discomfort caused to the patient. Extended use of electrocautery causes heat buildup in the tissues, which results in tissue destruction [7]. 

The soft tissue trimming bur abrasion method is a way to de-epithelialize the gingival epithelium layer by rapidly spinning a ceramic soft tissue trimming bur. Compared to other surgical techniques, gingival ablation with a diamond bur is a straightforward and efficient process that doesn't require complicated or durable instruments [1]. It was discovered that this technique had trouble regulating the de-epithelialization depth. Additionally, bleeding and pain following surgery are anticipated [8]. 

Recurrence of pigmentation is seen spontaneously and is found to be linked to the migration and activity of nearby cells containing melanocytes [9]. Although the exact mechanism of re-pigmentation remains unclear, the theory of migration suggests that re-pigmentation occurs as a result of melanocytes which are active and migrating to the treated areas from nearby tissues with pigmentation [10].

To date, there are no such studies comparing depigmentation by using an electrosurgical unit and soft tissue trimming bur in a split-mouth design; hence, this study was planned. The objective of this case study was to evaluate the difference in pain felt by the patient between the sites treated with electrosurgery and bur abrasion method and also to evaluate the healing of the surgical site by both methods.

## Case presentation

**Figure 1:**
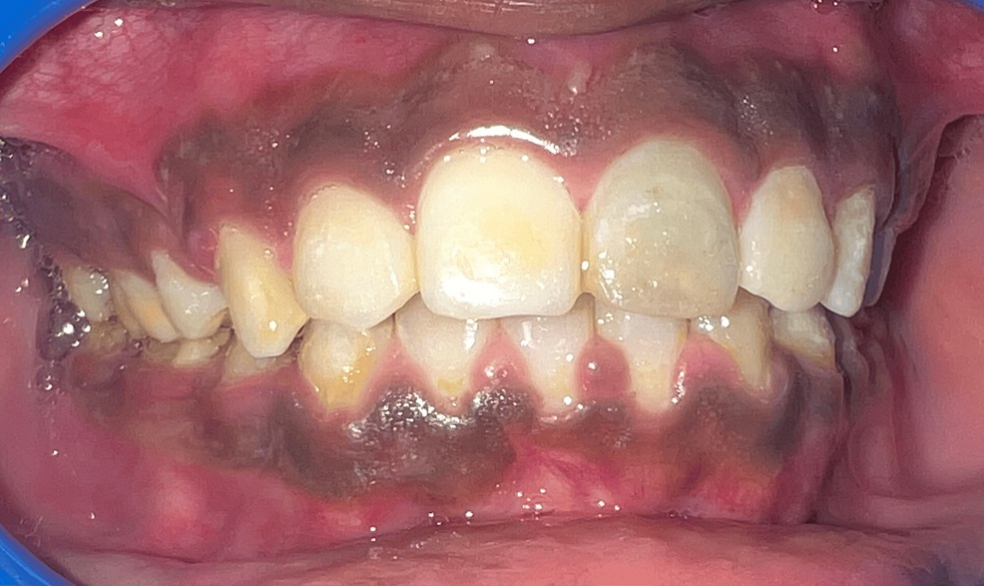
Pre-operative photograph showing pigmented gingiva

A 19-year-old male patient reported to the Department of Periodontics with heavily pigmented gums (Figure 1). Non-contributory medical history was obtained. There was no history of smoking or any adverse habit. On intraoral examination, gingiva with melanin pigmentation with a Dummett-Gupta oral pigmentation index (DOPI) score of 3 was seen, which did not show any inflammation. Different treatment options were explained, taking into account the patient's aesthetic concerns.

The whole of the maxillary arch was parted into two sections. Section I extended from the right canine to the right central incisors. Section II extended from the left canine to the left central incisors. These two sections were taken into account in a split-mouth design. Electrocautery was planned on the right side while bur abrasion therapy was planned on the left side. A blood investigation was done to make sure that surgery was not contraindicated. Oral prophylaxis practices were done and oral hygiene reinforcement was provided keeping the poor oral hygiene of the patient in mind. The entire procedure was explained to the patient and written consent was taken. 

Local anesthetic containing 2% lidocaine with 1:100000 epinephrine was infused into the maxillary anterior region under the topical anesthetic spray, extending from the canine to the canine.

Depigmentation using electrocautery

**Figure 2:**
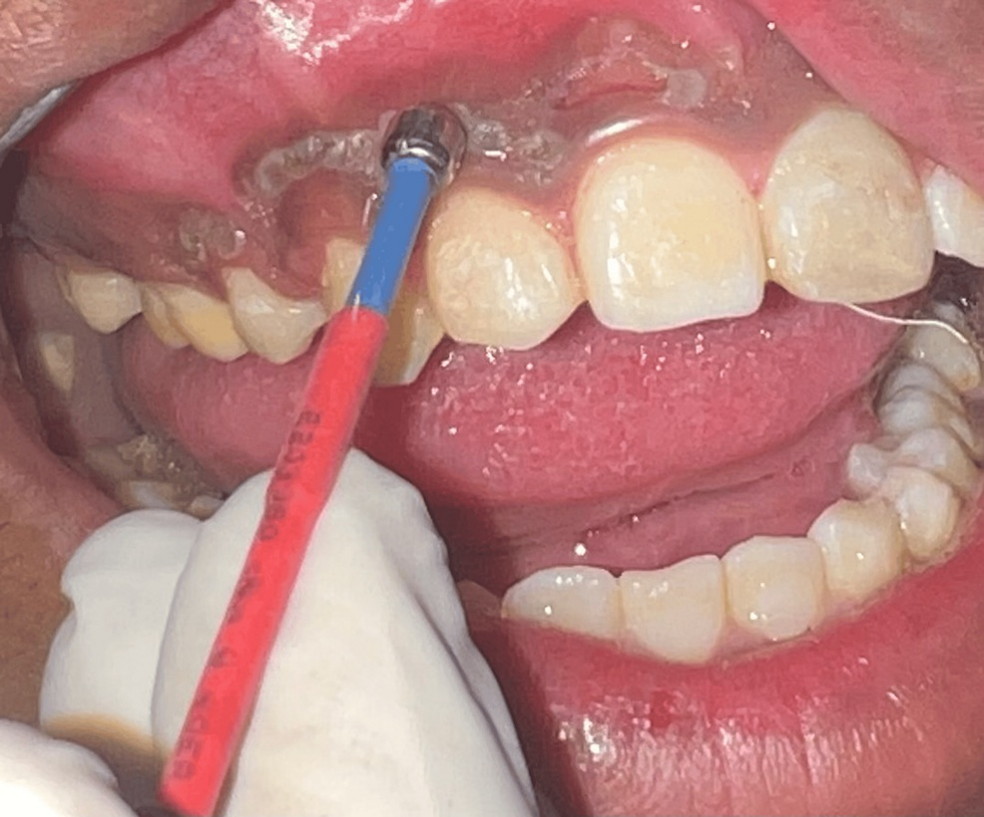
Operative view using electrocautery (Right)

**Figure 3:**
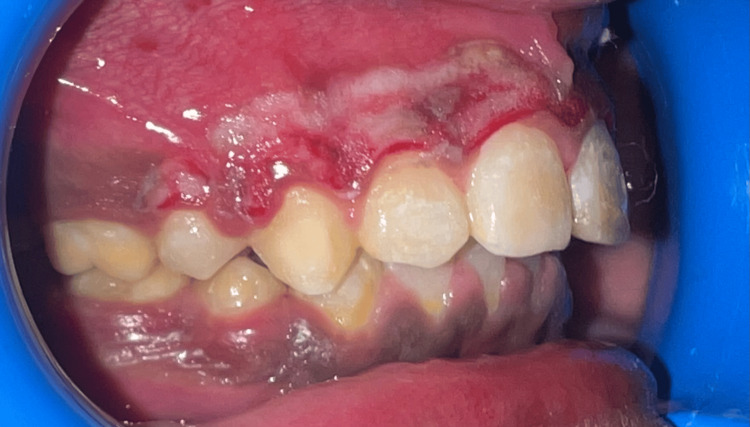
Immediate view after electrosurgery

Figure 2 shows depigmentation using electrocautery. A heavy bulky electrode was used for depigmentation on the right side. The clinically noticeable pigmentation was carefully removed using an electrosurgical unit (Figure 3), protecting the gingival contours, protecting the periosteum, and preventing tooth damage or exposure of the alveolar bone beneath a thin layer of connective tissue. In order to prevent heat buildup and tissue destruction, the tip was kept constantly moving. A periodontal dressing (COE-PAK™; GC International AG, Luzern, Switzerland) was applied to the depigmented area.

Depigmentation using soft tissue trimming bur

**Figure 4:**
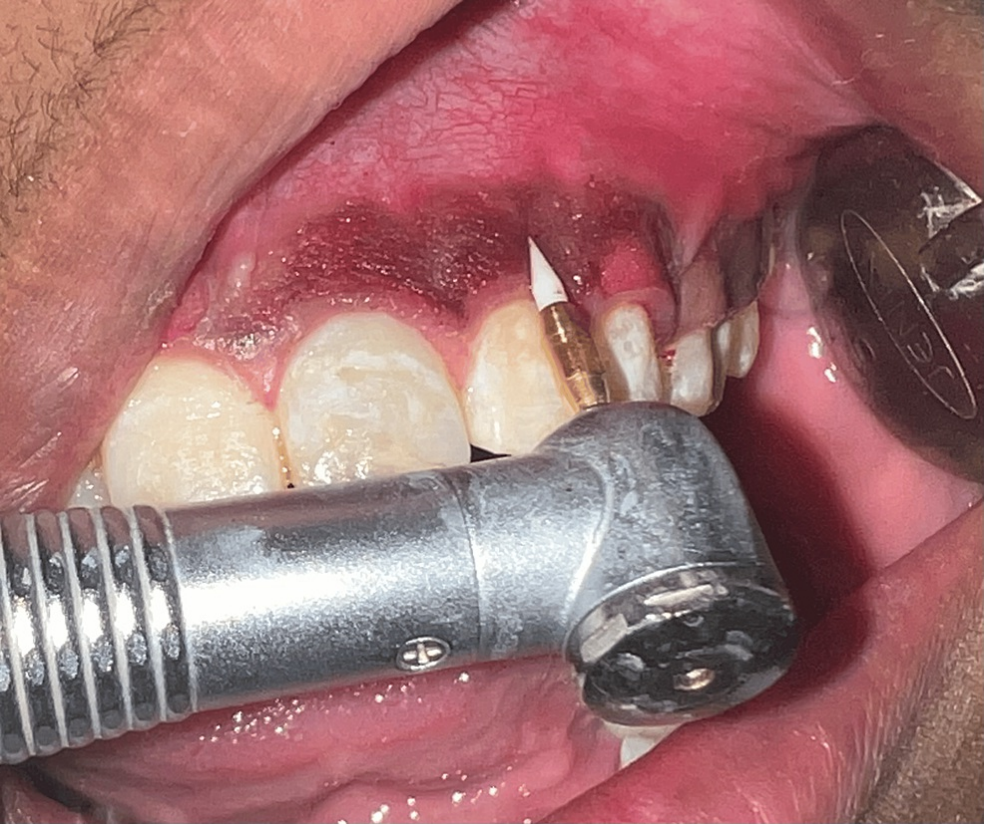
Operative view using Bur abrasion (left)

Soft tissue trimming bur rotated at high-speed rpm without the use of water coolant was utilized for depigmentation on the left segment for excising and contouring of gingival tissue (Figure 4). The heat production by the bur is due to friction, which results causes an immediate coagulation of tissues as well as minimal bleeding, and for this reason, coolant was not used.

**Figure 5:**
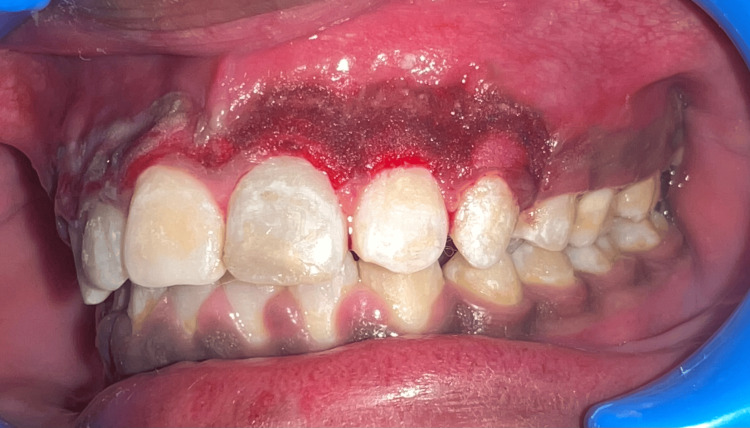
Immediate view after Bur abrasion therapy (Left)

**Figure 6:**
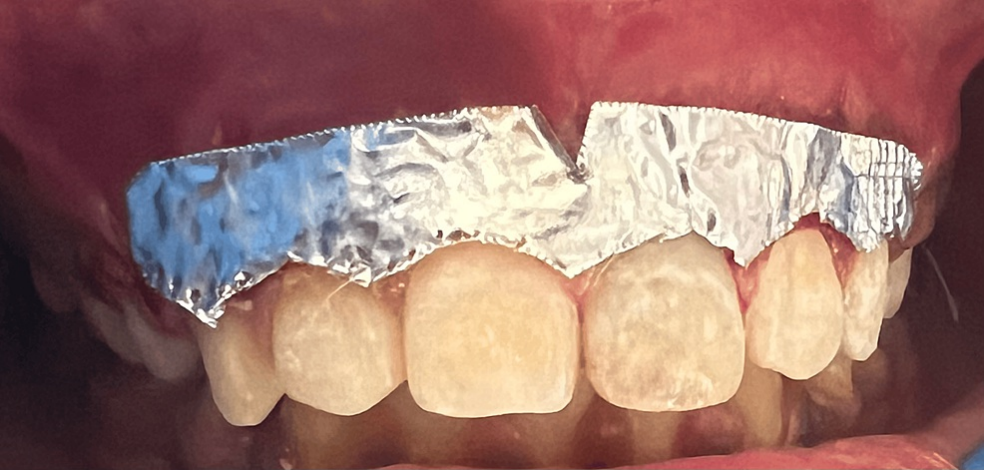
Tin foil placed over surgical site

**Figure 7:**
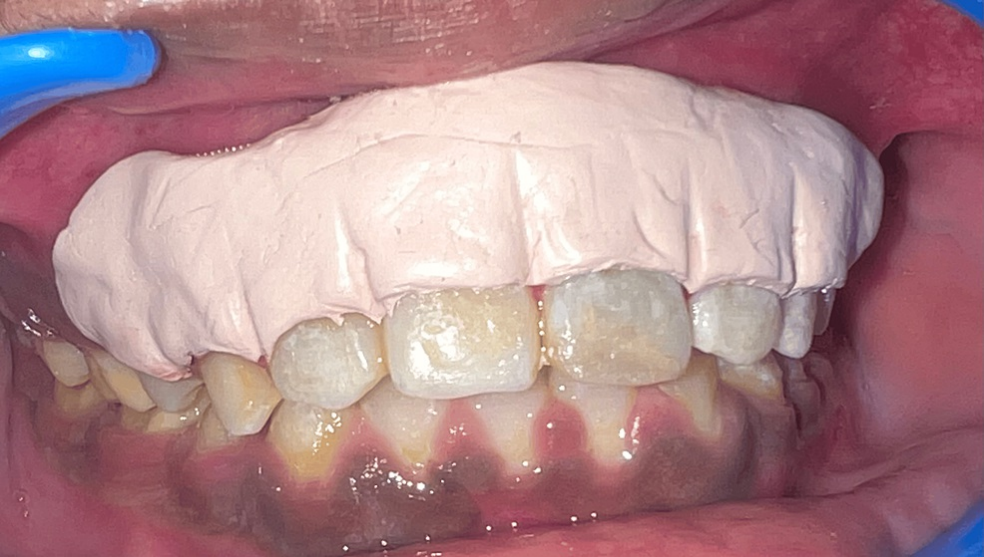
Surgical site covered with COE-PAK* COE-PAK™, GC International AG, Luzern, Switzerland

After the removal of the whole epithelium, which was pigmented, with the help of precision soft tissue trimming bur (Figure 5), saline irrigation was done on the exposed surface. It was taken into account that the entire remnants of the pigmented epithelial layer had been removed. Tin foil was placed over the surgical site (Figure 6). COE-PAK was placed over the surgical area (Figure 7).

**Figure 8:**
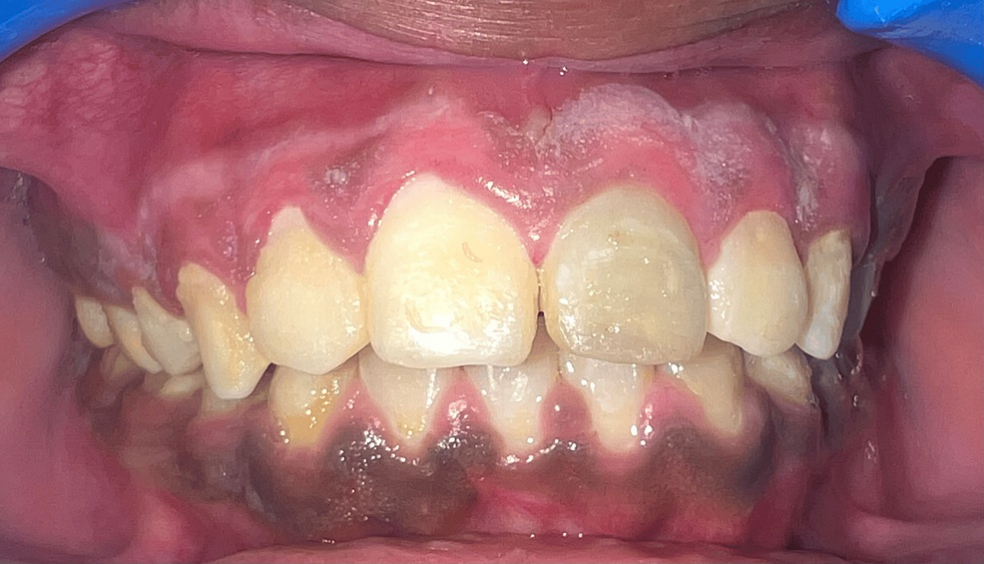
Post-operative picture after one week

**Figure 9:**
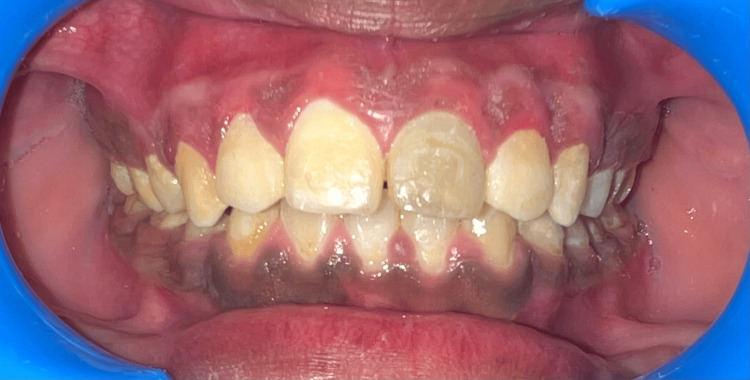
Post-operative picture after one month

Postsurgical instructions were given to the patient. The follow-up visit was kept at an interval of one week. The dressing was removed after one week, and the evaluation of the surgical area was done carefully (Figure 8). Upon a one-month follow-up (Figure 9), the healing process was seen to have proceeded without any post-operative complications.

The gingiva had a normal appearance, appearing pink, firm, and healthy. The mandibular anterior region was not treated with depigmentation because the patient did not find it to be an aesthetic concern. The following clinical parameters were evaluated: the postsurgical healing on the seventh day and at the end of one month, visual analog scale (VAS) to evaluate pain on the seventh day. Healing was assessed visually and it was better on the seventh day for the electrosurgical site as compared to the site treated with bur. At one month, visually both the groups showed complete healing. On the seventh day, the electrosurgical site showed mild clinical pigmentation as compared to heavy pigmentation before treatment. There was a VAS score of 3 which is mild pain felt by the patient at the bur abrasion site whereas a VAS score of 1 was given for the electrosurgical site on the seventh day.

## Discussion

Gingival color varies greatly amongst normally healthy individuals. The gingiva's color can be based on the vascularization degree, keratinized layer thickness, and the quantity of cells that contain pigment [11]. Basically, a layer containing connective tissue that underlines along with the gingival epithelium is surgically removed, and the denudement of the connective tissue is left which heals by secondary intention [12]. There is no pigmentation of melanin in the newly formed epithelium.

This case demonstrated that electrocautery treatment, as opposed to soft tissue trimming bur in the first and second weeks, promotes a faster rate of healing. When diamond burs are used for gingival depigmentation, the tissue injury which results is more difficult to control, resulting in an uneven, surface that is not smooth and that takes a longer time to heal [6]. This may result in blood and lymph fluids seeping through, causing swelling to last longer. As electrocautery shows adequate hemostasis and proper incision margin placement, the inflammatory phase is also accelerated and the swelling is reduced. These factors play an important part in improving the rate of healing. The cross-sectional test results demonstrated a statistically significant difference in pain during the procedures comparing the two treatments. There was more pain experienced by the patient for the bur site on the first day. 

In this study, healing was visually assessed and it was better and faster on the seventh day in the electrosurgical site as compared to the site treated with soft tissue bur. At the one-month follow-up, it was complete for both groups. Significant improvement was seen in gingival color on the seventh day in the electrocautery site with mild pigmentation as compared to heavy pigmentation before treatment. A VAS score of 3, which is mild pain, was given for the bur-treated site, and for the electrosurgical site, a VAS score of 1 was given by the patient on the seventh day.

Histopathological analysis should be done to evaluate the healing of the surgical site, which wasn't done in the current case. 

## Conclusions

Depending on whether gingival pigmentation is pathological or physiological, it varies. Patients' psychological needs, along with the treatment acceptance and aesthetic outcome, are the most crucial factors that determine the treatment option for gingival melanin hyperpigmentation. Older techniques like electrocautery, cryotherapy, laser procedures, bur abrasion, and gingival abrasion by scalpel surgery can all be used to treat melanin pigmentation. When gingival hyperpigmentation is treated with a diamond bur, the healing process takes longer than when electrocautery is used. The treatment site with the diamond bur had more pain on the first day following the procedure. The electrocautery-treated site in the present case showed better results in terms of pain experienced by the patient, and the surgical site healing was also faster and better.
